# Process intensification at the expression system level for the production of 1-phosphate aldolase in antibiotic-free *E. coli* fed-batch cultures

**DOI:** 10.1093/jimb/kuac018

**Published:** 2022-06-06

**Authors:** Martina Pasini, Alfred Fernández-Castané, Gloria Caminal, Tim W Overton, Pau Ferrer

**Affiliations:** Aston institute of Photonic technologies (AiPT), Aston University, Birmingham, B4 7ET, UK; Department of Chemical, Biological, and Environmental Engineering, Escolad'Enginyeria, Universitat Autònoma de Barcelona, Bellaterra (Cerdanyola del Vallès) 08193, Catalonia, Spain; Aston Institute of Materials Research, Aston University, Birmingham, B4 7ET, UK; Energy and Bioproducts Research Institute, Aston University, Birmingham, B4 7ET, UK; Department of Chemical, Biological, and Environmental Engineering, Escolad'Enginyeria, Universitat Autònoma de Barcelona, Bellaterra (Cerdanyola del Vallès) 08193, Catalonia, Spain; Institute of Advanced Chemical of Catalonia, IQAC−CSIC, 08034, Barcelona, Spain; School of Chemical Engineering, College of Engineering and Physical Sciences, University of Birmingham, Edgbaston, Birmingham, B15 2TT, UK; Institute for Microbiology and Infection, University of Birmingham, Edgbaston, Birmingham, B15 2TT, UK; Department of Chemical, Biological, and Environmental Engineering, Escolad'Enginyeria, Universitat Autònoma de Barcelona, Bellaterra (Cerdanyola del Vallès) 08193, Catalonia, Spain

**Keywords:** High-cell-density fed-batch cultures, Recombinant protein production, Antibiotic-free expression system, *Escherichia coli*, Bioprocess optimization

## Abstract

To successfully design expression systems for industrial biotechnology and biopharmaceutical applications; plasmid stability, efficient synthesis of the desired product and the use of selection markers acceptable to regulatory bodies are of utmost importance. In this work we demonstrate the application of a set of IPTG-inducible protein expression systems -- harboring different features namely, antibiotic vs auxotrophy marker; two-plasmids vs single plasmid expression system; expression levels of the repressor protein (LacI) and the auxotrophic marker (glyA) -- in high-cell density cultures to evaluate their suitability in bioprocess conditions that resemble industrial settings. Results revealed that the first generation of engineered strain showed a 50% reduction in the production of the model recombinant protein fuculose-1-phosphate aldolase (FucA) compared to the reference system from QIAGEN. The over-transcription of glyA was found to be a major factor responsible for the metabolic burden. The second- and third-generation of expression systems presented an increase in FucA production and advantageous features. In particular, the third-generation expression system is antibiotic-free, autotrophy-selection based and single-plasmid and, is capable to produce FucA at similar levels compared to the original commercial expression system. These new tools open new avenues for high-yield and robust expression of recombinant proteins in E. coli.

## Introduction

*Escherichia coli* is the most widely used host for the development of bacterial expression systems for overexpression of recombinant proteins and remains the first choice for laboratory investigations and industrial production of recombinant proteins within the biopharmaceutical sector (Rosano & Ceccarelli, 2014; Castiñeiras et al., 2018). The main advantages of *E. coli* are rapid growth rate, capability to reach high cell densities, growth on inexpensive substrates, well-characterized genetics, and the availability of excellent tools for genetic manipulation (Brown, 1995).

A wide range of expression systems with different features have been developed for recombinant protein production (RPP) in *E. coli*, comprising plasmids for constitutive or inducible expression, different mechanisms of induction (e.g., temperature and IPTG-inducible systems), the use of strains with different genotypes (Waegeman & Soetaert, 2011), and additional features to aid production (e.g., use of tuneable strains or co-expression of chaperones; Marschall et al., 2017). Moreover, expression systems may incorporate fusion tags to increase protein solubility or enable purification using affinity chromatography (Costa et al., 2014). The vast majority of expression systems for lab-based investigations include antibiotic resistance markers that enable screening of positive clones and help in plasmid retention. However, the use of expression systems with antibiotic markers is considered unacceptable by regulatory authorities in relevant areas of industrial biotechnology (e.g., medical, therapeutic, and agricultural applications; Glenting & Wessels, 2005) and follows current trends in improvements in antibiotic use and stewardship to reduce antimicrobial resistance (Mignon et al., 2015).

In order to produce high levels of protein, it is often useful to clone the gene of interest downstream to a well-characterized, strong, and tightly regulated promoter. The *E. coli lac* promoter is arguably the most extensively studied (Germán et al., 2019). Within the realm of *E. coli* expression, the T5 promoter system (and its derivatives) available from QIAGEN is one of the most popular choices for protein production (Fernández-Castané et al., 2012a; Calleja et al., 2014). This IPTG-induced promoter has a high transcription rate that can only be tightly regulated in the presence of high levels of the Lac repressor protein (LacI), which is encoded by the *lacI* gene (Xu & Matthews, 2009). The commercial pQE- vectors commercialized by QIAGEN utilize two *lac* operator (*lacO*) regions in order to guarantee strong repression under non-induced conditions (Rosano & Ceccarelli, 2014).

It is widely recognized that RPP consumes cellular energy and metabolites (Rahmen et al., 2015), whereby high level expression of heterologous proteins has a direct impact on the central metabolism of cells and causes the activation of stress responses (Weber et al., 2002; Neubauer et al., 2003). This physiological response is known as the metabolic burden caused by RPP draining the host cell's resources, either in the form of energy such as ATP or GTP, or nutrients such as amino acids and nucleotides that are required to express and synthesize the protein of interest and its associated cellular machinery (Glick, 1995). Typically, cells overcome the metabolic burden by triggering stress-response mechanisms that adapt and readjust the metabolism in order to restore functionality and viability. However, such cellular responses often negatively affect growth parameters such as growth rate, biomass yield, and cellular viability, as well as recombinant protein productivity (Carneiro et al., 2012).

Plasmid-based expression systems are not always stable, particularly in cultivation strategies where cells are grown for many generations, that is, in high-cell-density or continuous cultures (Pierce & Gutteridge, 1985). Hence, vector stability is of utmost importance. Expression systems using two or more plasmids may present high instability, thus enabling their use only as ‘‘transient’’ expression systems (Al-Allaf et al., 2013). During cell growth in high-cell-density cultures, overexpression of recombinant proteins often results in (i) cell aggregation and (ii) bacteria becoming viable but non-culturable, a phenotype which means the loss of the ability to form colonies on agar plates (Jeong & Lee, 2003; Wyre & Overton, 2014a; Fernández-Castané et al., 2017). Flow cytometry (FCM) has been extensively used as a rapid and high-throughput analytical technique to determine the viability and physiology of cells and to detect heterogeneities in cultures (Hewitt et al., 1999; Wyre and Overton, 2014b).

Previous studies carried out in our laboratory developed auxotrophic selection systems based on glycine auxotrophy derived from the commercial two-plasmid pQE-based expression system (QIAGEN), comprising an expression plasmid (pQE), where the T5 promoter regulates expression of the recombinant gene of interest, and pREP4, which expresses *lacI* that allows tight regulation of the T5 promoter on the pQE plasmid (Gronenborn, 1976). The *E. coli glyA* gene encoding serine hydroxymethyl transferase (SHMT), which catalyzes the reversible interconversion of L-threonine and glycine and is essential for cell growth in the absence of glycine (Plamann & Stauffer, 1983), was used as a selective marker. In the first generation auxotrophic system (Vidal et al., 2008), *glyA* was cloned into the pQE-FucA vector expressing *fucA*, encoding fuculose-1-phosphate aldolase (FucA). The resultant plasmid, pQEαβFucA, was transformed into the glycine auxotroph *E. coli* M15*ΔglyA*[pREP4] yielding M15*ΔglyA*[pREP4] pQEαβFucA. Expression studies in shake flask cultures revealed that this strain presented a slight decrease in recombinant protein yield compared to the commercial reference strain (Pasini et al., 2016).

In the second generation of the expression system (named ‘‘Puzzle’’), we optimized expression levels of *lacI* and *glyA* using the weak, constitutive J23110 synthetic promoter (Registry for Standard Biological Parts, http://parts.igem.com) and cloned both as cassettes into the pQE-FucA vector, which was transformed into *E. coli* M15Δ*glyA*, removing the need for the pREP4 plasmid and thus alleviating the metabolic burden. This resulted in improved plasmid maintenance while substantially increasing the production levels of the protein of interest in shake flasks experiments (Pasini et al., 2016). Finally, the third generation strain (‘‘AmpR^–^’’) was generated by deleting the ampicillin resistance gene (*bla*) from the pQE-FucA plasmid, resulting in solely auxotrophic selection.

In this work, we demonstrate that the third generation of antibiotic-free pQE-derived expression systems developed recently in our laboratory can be successfully used in industrially relevant bioprocessing strategies (i.e., in high-cell-density cultures) for the expression of FucA, an enzyme of interest for biocatalytic pharmaceutical applications (Koeller & Wong, 2000; Clapés et al., 2010) . Growth and production of FucA were compared in the reference and three generations of auxotrophic systems, and cell physiology was measured using FCM. Our third generation system is advantageous as it does not require antibiotics and comprises a single plasmid.

## Materials and Methods

### Strains and Plasmids

The K-12 derived *E. coli* M15 (QIAGEN) and M15*ΔglyA* strains were used for recombinant FucA expression. These strains were stored at –80°C in cryo-stock aliquots prepared from exponential phase grown cultures in Luria-Bertani (LB) medium. The list of *E. coli* strains, plasmids, and the expression systems used in this study and their abbreviated names are summarized in Table 1.

**Table 1.** Bacterial strains and plasmids used in this work and abbreviation for the four *E. coli* expression systems used in this study

Name	Characteristics	Reference or source	
*Strains*			
M15	K12 derived	QIAGEN (Zamenhof & Villarejo, 1972; Villarejo & Zabin 1974)	
M15*ΔglyA*	Deletion of the *glyA* locus of the chromosome	Vidal et al. (2008)	
*Plasmids*			
pQE-FucA	pQE-40 derived (QIAGEN) with *fucA* gene cloned	Pasini et al. (2016)	
pREP4	LacI^q^ kan^R^	QIAGEN	
pQEαβFucA	pQE-FucA derived with fragment, containing the *glyA* gene, under the P3 promoter transcriptional control	Pasini et al. (2016)	
pQE-FucA_puzzle	pQE-FucA derived with insertion of the cassette (J23110-*lacI*-*glyA*) for *lacI* and *glyA* genes transcription	Pasini et al. (2016)	
pQE-FucA_puzzle_AmpR^–^	pQE-FucA_puzzle (J23110) derived without the *bla* gene	Pasini et al. (2016)	
Expression system name	Abbreviation	Generation	Number of plasmids
M15[pREP4] pQE-FucA	M15[pREP4]	Reference system	2
M15*ΔglyA*[pREP4] pQEαβFucA	M15*ΔglyA*[pREP4]	First generation system	2
M15Δ*glyA* pQE-FucA_puzzle (J23110)	Puzzle	Second generation system	1
M15*ΔglyA* pQE-FucA_puzzle (J23110)_AmpR^–^	AmpR^–^	Third generation system	1

### Culture Media

All chemicals and reagents were purchased from Sigma-Aldrich unless otherwise indicated. Luria-Bertani medium, containing 10 g L^–1^ peptone, 5 g L^–1^ yeast extract, and 10 g L^–1^ NaCl, was used for pre-cultures.

Defined medium (DM) used for shake flasks cultures and bioreactors contained per liter: 5 g glucose; 2.97 g K_2_HPO_4_; 0.60 g KH_2_PO_4_; 0.46 g NaCl; 0.75 g (NH_4_)_2_SO_4_; 0.11 g MgSO_4_·7H_2_O; 0.006 g FeCl_3_; 0.025 g thiamine; 1.44 g CaCl_2_·2H_2_O; and 0.7 mL of trace elements solution (TES). TES contained per liter: 0.04 g AlCl_3_·6H_2_O; 1.74 g ZnSO_4_·7H_2_O; 0.16 g CoCl_2_·6H_2_O; 2.18 g CuSO_4_·5H_2_O; 0.01 g H_3_BO_3_; 1.42 g MnCl_2_·6H_2_O; 0.01 g NiCl_2_·6H_2_O; and 0.23 g Na_2_MoO4·5H_2_O.

Stock solutions of kanamycin and chloramphenicol were dissolved in dH_2_O at a concentration of 100 mg mL^–1^ and 30 mg mL^–1^, respectively and stored at –20 °C. Ampicillin was dissolved in absolute EtOH and prepared at a concentration of 100 mg L^–1^ and stored at –20 °C. IPTG stock was prepared at 100 mM, and stored at –20 °C.

Vitamins, antibiotics, TES, FeCl_3_, MgSO_4_·4H_2_O, CaCl_2_·2H_2_O, and inducer were sterilized by filtration (0.2 μm syringe filter made from a blend of cellulose esters, Sartorius). Glucose and saline solutions were separately sterilized by autoclaving at 121°C for 30 min.

Feeding media for the fed-batch phase contained per litre: 490 g glucose; 9.56 g MgSO_4_·7H_2_O; 0.49 g FeCl_3_; 0.33 g thiamine; 0.10 g CaCl_2_·2H_2_O; and 63 mL of TES. Phosphates (P) were not included in the feeding solution in order to avoid co-precipitation with magnesium salts. Instead, a 5-mL pulse of a concentrated phosphates solution containing 500 g L^−1^ K_2_HPO_4_, and 100 g L^−1^ KH_2_PO_4_ was added every 30-OD_600_ step increase based on a calculated biomass from phosphates yield (*Y*_X/P_) of 18 g DCW g P^–1^ (Vidal et al., 2003).

### Cultivation Conditions

Cryo-stocks stored at –80°C, were used to inoculate 50 mL Falcon tubes with 15 mL of LB medium supplemented with the corresponding antibiotic when necessary. Growth was performed overnight at 37 °C at 200 rpm.

A total of 3 mL of overnight pre-inoculum were transferred into a 500 mL conical flask containing 100 mL of DM, following the same growing conditions as pre-inoculum cultures for approximately 4.5 hr until an OD_600_ of 1.1–1.2 was achieved.

Bioreactor experiments were carried out using two types of stirred tank bioreactors: Braun Biotech Int. Biostat® B (Germany) and Electrolab Fermac 310/60 (Tewkesbury, UK). For both bioreactors, the temperature was maintained at 37°C and the pH was kept at 7.00 ± 0.05 by adding 15% (v/v) NH_4_OH solution (NH_4_^+^ from base addition served also as a nitrogen source). Dissolved oxygen level (pO_2_) was kept above 60% saturation. After glucose was depleted during the batch phase, feeding was started with the addition of the feed solution.

Induction of cultures was carried out by a single-pulse of 70 μM IPTG as previously described by (Fernández-Castané et al., 2012b). During the induction period, 1.5 mL samples were removed from the bioreactor every 10–30 min intervals. One sample was removed prior to induction for the measurement of basal FucA expression.

For experiments performed in the Biostat® B bioreactor, a volume of 80 mL of the inoculum culture was added to 720 mL of DM in a 2-L vessel (Vidal et al., 2005; Calleja et al., 2014). pO_2_ set point was fixed at 60% saturation, and pO_2_ was controlled by adapting the stirring speed between 350 and 1120 rpm and by supplying air (enriched with pure oxygen when necessary) at a flow rate of 1.5 vvm. A microburette was used for the discrete addition of feed. The pH was set at 7 and maintained by adding a 15% (v/v) NH4OH solution.

For experiments performed in the Fermac 310/60 bioreactor, the volume of inoculum was 200 mL and the DM volume was 1300 mL, making up a total of 1500 mL batch volume in a 5-L jar equipped with 4 baffles and an agitator with 2 six-bladed Rushton turbines. Aeration was achieved by sparging air from below the lower impeller at a rate of 3 L hr^–1^ (2 vvm). pO_2_ was measured online using a D150 Oxyprobe (Broadley James) and was maintained above a set point of 50% by increasing agitation to a maximum of 1000 rpm from a minimum of 200 rpm. Off-gas passed through a condenser, an autoclavable 0.22-μm filter (Sartorius, Goettingen, Germany), and a HEPA filter (Millipore, Darmstadt, Germany). pH was measured using an F-695 FermProbe (Broadley James). The temperature was maintained at 37 °C by a heating jacket and a cold finger. The pH was set at 7 and maintained by adding a 15% (v/v) NH4OH solution.

Bioreactor off gasses were automatically collected for subsequent compositional analysis using a PrimaDB gas-chromatograph mass spectrometer (Thermo) and compared to atmospheric air in order to calculate the O_2_ uptake rate, CO_2_ evolution rate, and subsequent estimation of the respiratory quotient (RQ). These data were logged automatically by GasWorks v1.0 (Thermo). The increase of pO_2_, O_2_, and pH values, together with a decrease in CO_2_ values, indicate the complete consumption of the 10 g L^–1^ of initial glucose and the end of the batch phase (Supplementary data, Fig. S2-1), (Sooan at al., 2009).

The feeding strategy was performed to maintain the specific growth rate (*μ*_fix_) constant through the fed-batch phase, using a pre-defined exponential feeding profile (Pinsach et al., 2008). Feeding was manually and periodically interrupted and restarted in order to avoid glucose accumulation during the induction phase.

### Analytical Methods

Cell concentration was determined by optical density measurements at 600 nm (OD_600_) using a spectrophotometer (Uvicon 941 Plus, Kontrol, and Evolution 300 UV–vis, Thermo Scientific, for the Biostat® B and the Fermac 310/60 bioreactors, respectively). OD_600_ values were correlated to biomass concentration expressed as Dry Cell Weight (DCW), being 1 OD_600_ equivalent to 0.3 gDCW L^–1^ (Pinsach et al., 2008).

Glucose and acetate concentrations in the fermentation broth were analyzed. One milliliter of culture medium was separated from biomass by centrifugation at 14 000 rpm for 6 min and filtered (0.45 μm membrane filter of cellulose esters, Millipore) prior to analysis. Glucose concentration was determined enzymatically using an YSI 2070 biochemical analyzer (Yellow Spring Systems). Acetic acid was analyzed by HPLC (Hewlett Packard 1050) equipped with an ICSep COREGEL 87H3 ICE-99-9861 (Transgenomic) column and an IR detector (HP 1047), using 6 M H_2_SO_4_ (pH 2.0) as the mobile phase at a flow rate of 0.3 mL min^–1^. The column was kept at 40°C.

The maximum specific growth rate (μ_max_) of the strains was estimated from microbial growth curves. *Equation 1* shows the relationship between the cell concentration (*X*), maximum specific growth rate (μ_max_), and time (*t*). Log-linearized *Equation**2* yields a linear relationship, where the μ_max_ is represented by the slope of the linear portion in the plot of the natural log of cell concentration versus time.

(1) \begin{equation*} {X}_t = {X}_0\ \cdot {e}^{{\mu }_{{\rm{max}}} \cdot t} \end{equation*}

(2) \begin{equation*} {\rm{ln}}{X}_t = {\rm{ln}}{X}_0\ + {\mu }_{{\rm{max}}} \cdot t, \end{equation*}

where *X_0_* and *X_t_* are the OD_600_ or cell concentration at the start and during the exponential phase of growth.

The biomass yield, *Y_X/S_* was calculated using the following equation:

(3) \begin{equation*} {Y}_{X/S} = \frac{{({\rm{DC}}{{\rm{W}}}_{{\rm{max}}} - {\rm{DC}}{{\rm{W}}}_0)}}{{({\rm{Gl}}{{\rm{c}}}_0 - {\rm{\ Gl}}{{\rm{c}}}_{\rm{f}})}}\ . \end{equation*}

DCW_max_ and DCW_0_ (g L^−1^) are the maximum and the initial biomass values, respectively. Glc_0_ and Glc_f_ (g L^−1^) are initial and final value of glucose concentration, respectively.

### FucA Quantification

Samples from fermentation broth were withdrawn, adjusted to a final OD_600_ of 4, centrifuged, and subsequently processed as described elsewhere (Durany et al., 2005; Vidal et al., 2008). Briefly, pellets were resuspended in 100 mM Tris HCl (pH 7.5). Cell suspensions were placed in ice and sonicated [Vibracell^TM^ model VC50 (Sonics & Materials)], over four pulses of 15 s each at 50 W with 2 min intervals in ice between each pulse. Cellular debris was then removed by centrifugation, and the cleared supernatant was collected for FucA analysis. One unit of FucA activity is defined as the amount of enzyme required to convert 1 μmol of fuculose-1-phosphate in DHAP and L-lactaldehyde for minute at 25 °C and pH 7.5 (Vidal et al., 2008).

Total protein content was determined by means of the Bradford method using a Coomassie® Protein Assay Reagent Kit (Thermo Scientific). To quantify the amount of FucA relative to total intracellular soluble proteins, NuPAGE® 12% Bis–Tris gels were used according to the manufacturer's instructions (Invitrogen). Protein concentration was quantified using Kodak Digital Science® 1D 3.0.2 densitometry software.

Average values of triplicates experiments were plotted with error bars. The error indicates the confidence interval of 90%.

### Flow Cytometry

Bacterial samples taken directly from the bioreactor were resuspended in phosphate-buffered saline to a final concentration of 10^5^–10^6^ cells mL^–1^ and then analyzed directly or after staining with various fluorescent dyes using a BD Accuri^TM^ C6 flow cytometer (Becton, Dickinson and Company, Oxford, UK). Samples were stained with bis (1,3-dibutybarbituric acid) trimethine oxonol (0.05 μg BOX mL^–1^) to determine membrane potential; propidium iodide (4 μg PI mL^–1^) to determine membrane integrity; or Congo red (CR) (40 μg CR mL^–1^) to determine amyloid content and thus the presence of inclusion bodies (IBs). During FCM on fluorescently labeled cells, samples were excited using a 488 nm solid-state laser, and fluorescence was detected using two different filters: a 533/30 BP (filter FL1-A) for BOX; and a 670 LP filter (FL3-A) for PI and CR. Particulate noise was eliminated using a FSC-H threshold. A total of 20 000 data points were collected at a maximum rate of 2500 events s^–1^. Data were analyzed using BD CFlow software.

## Results

### Comparison of Cell Growth, Substrate Consumption, and Acetate Formation

The performance of the three generations of auxotrophic expression systems (Table 1) was compared to that of the initial QIAGEN pQE/pREP4 system in fed-batch fermentations for the production of FucA. These strains had previously only been compared in shake flask cultures (Pasini et al., 2016). In this study, triplicate fed-batch fermentations were performed using defined medium and glucose as a carbon source in BioStat bioreactors to determine the robustness of each expression system in terms of growth yield, plasmid stability, and RPP.

**Fig. 1.**
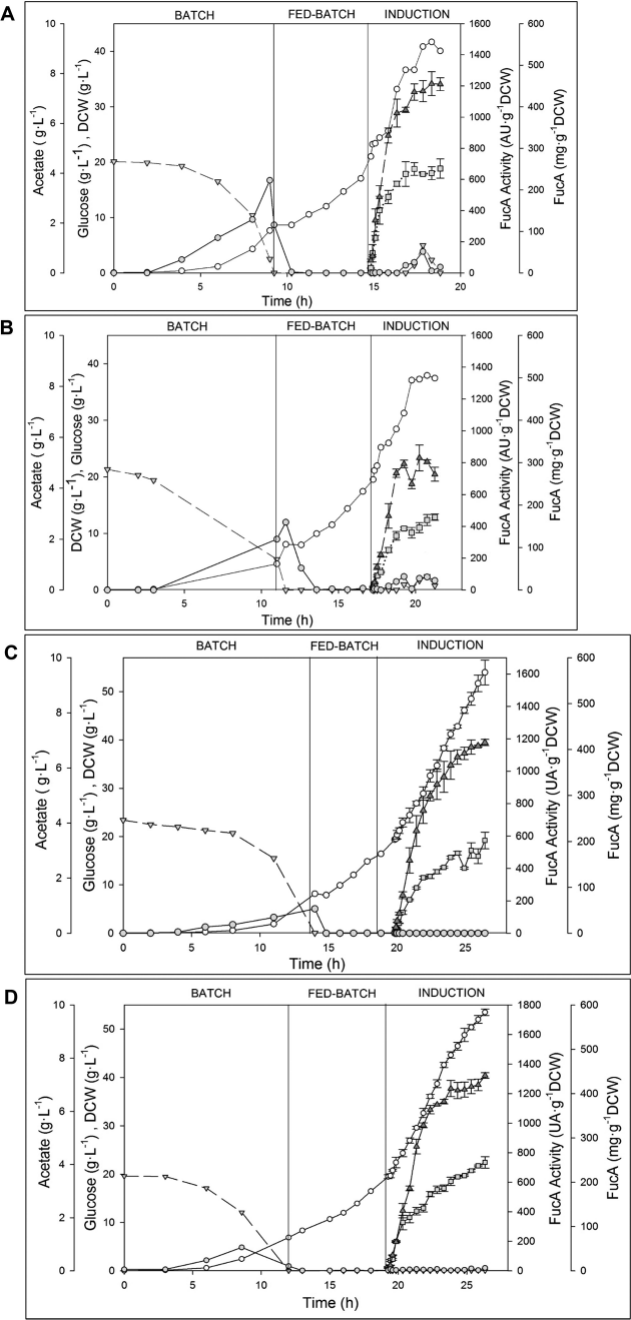
Time profiles of fed-batch cultures of FucA production strains performed under the following conditions: [IPTG], 70 μM; *X*_ind_, 20 g · L^–1^; *μ*_fix_, 0.22 hr^–1^. (A) Reference strain, M15[pREP4] pQE-FucA; (B) first generation, M15Δ*glyA*[pREP4]; (C) second generation, Puzzle; (D) third generation, AmpR^–^. Key to symbols: (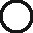) biomass DCW (g L^–1^); (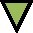) glucose concentration (g L^–1^); (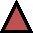) FucA activity (AU g^–1^ DCW); (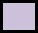) FucA specific mass (mgFucA ^–1^ DCW); and (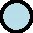) acetate concentration (g L^–1^). Batch, fed-batch, and induction phases are indicated. Triplicate fermentations were performed and mean ± SD are presented. The arrows indicate the stop of the feeding.

The time-profiles of the biomass, glucose consumption, FucA mass, specific activity, and acetate production of the four expression systems evaluated in this work are represented in Fig. 1. Key fermentation parameters are listed in Table 2.

**Table 2.** Calculated maximum specific growth rate and biomass from substrate yield of the *E. coli* strains used in this work along the batch phase

*Escherichia coli* strains	μ_max_ (hr^–1^)	*Y_x/s_* (g g^–1^)
M15[pREP4] (reference strain)	0.49 ± 0.02	0.43 ± 0.04
M15Δ*glyA*[pREP4] (first generation strain)	0.44 ± 0.01	0.36 ± 0.06
Puzzle (second generation strain)	0.45 ± 0.01	0.37 ± 0.09
AmpR^–^(third generation strain)	0.41 ± 0.01	0.35 ± 0.02

*Note*. Determination of FucA expression levels.

The comparison of the maximum specific growth rate (μ_max_) in the batch phase between the reference commercial M15[pREP4] *E. coli* strain and the three M15*ΔglyA*-derived generations shows that the latter present slightly lower μ_max_, decreasing from 0.49 ± 0.01 hr^–1^, of the reference strain to 0.44 ± 0.01 hr^–1^, 0.45 ± 0.01 hr^–1^, and 0.41 ± 0.01 hr^–1^ of the first, second, and third generations, respectively (Table 2). Furthermore, in both the reference and the first generation strains, the concentration of the biomass progressively increased until 2.5 hr after induction and then stopped around 4 hr after induction, reaching a final value of 40 g DCW L^–1^. While for the third generation strain the fermentation ended with a final biomass concentration of 53.5 ± 0.7 g DCW L^–1^, with a concentration of dissolved oxygen dropped below 10%, after 26.3 hr (Fig. 1).

Substrate uptake rates (q_S_) along the induction phase for both reference and the first generation strains were calculated, being 0.37 ± 0.04 and 0.50 ± 0.13 g Glc g^–1^ DCW hr^–1^, respectively. As a consequence, the first generation strain accumulated higher amounts of acetate throughout, reaching a final concentration of 0.54 ± 0.03 g L^–1^. Both strains started to accumulate glucose and acetate in the medium after 2 hr of induction, remaining below inhibitory levels (32 g L^–1^ and 2.4 g L^–1^, respectively) (Fig. 1A and B) (Kazan et al., 1995; Phue et al., 2005). It is widely known that glucose acts as a catabolite repressor, thus repressing the genes involved in metabolizing other sugars and, in IPTG-inducible systems, to repress the expression of recombinant proteins (Amma et al., 2018). The accumulation of glucose and acetate during the induction phase occurred at the same time as a decrease in the FucA activity and production in the first-generation system (Fig. 1B).

This accumulation is probably due to the high energy demand imposed to the cell for the synthesis of the recombinant protein. Recently, it has been demonstrated that this ‘‘metabolic burden’’ is not caused by energy limitation; on the contrary, this is due to restrictions in anabolic functions (Weber et al., 2021). This energy excess due to RPP causes a reduction of catabolic carbon processing, hence, (i) affecting negatively the growth rate; (ii) enhancing acetate formation, and (iii) reducing the carbon substrate uptake, evidenced by the accumulation of glucose (Weber et al., 2002, 2021).

The feeding strategy of our fed-batch cultivations was pre-set to maintain constant values of µ and *Y_X/S_*, and these were not modified during the induction phase, that is, the substrate feeding program assumed these parameters were constant even though they changed as a result of the metabolic imbalance generated by RPP, leading to an accumulation of glucose into the medium. When this state was reached, glucose feeding was manually stopped to avoid its further accumulation and re-established once the residual glucose was depleted.

Remarkably, the second and third generation strains (each with a single plasmid), unlike the previous two-plasmid systems, did not generate acetate or accumulate glucose during the induction phase (Fig. 1C and D). Thus, the tuning of *lacI*-*glyA* expression by the weaker constitutive J23110 promoter leads to a reduction in the transcription levels of these genes, resulting in a reduction of the metabolic load imposed to the cells.

**Fig. 2.**
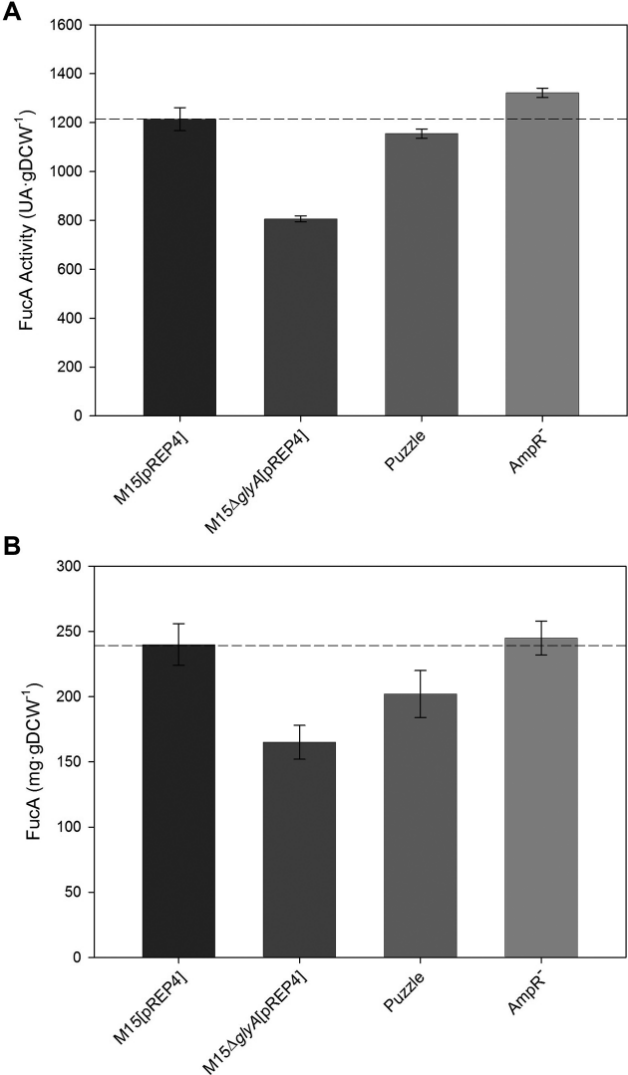
(A) Maximum enzyme activity (AU g^–1^ DCW) and (B) maximum specific mass (mgFucA g^–1^ DCW) for the strains presented in this study. The dashed line indicates the reference value corresponding to the reference (M15[pREP4]) strain.

In order to evaluate the levels of FucA production in fed-batch cultures of the different strains, specific protein production (mg g^–1^DCW) and activity (AU g^–1^ DCW) were measured as shown in Figs. 1 and 2, respectively. Overall, FucA activity and FucA production decreased more than 35% when comparing the first generation to the reference strain, which reached a final production of 240 ± 16 mg FucA g^–1^ DCW, with an activity of 1214 ± 47 AU g^–1^ DCW. These values were reduced to 165 ± 13 mg FucA g^–1^ DCW and 806 ± 12 AU g^–1^ DCW, respectively, in the first generation strain (Fig. 2). These effects may be caused by the increase in the metabolic burden due to the maintenance of the pQEαβFucA expression vector in the first generation auxotrophic strain. The *glyA* gene, even though encoded by the low-copy number plasmid (ColE1 replication origin) under the control of the P3 from the gen *bla*_TEM-1A_ (Lartigue et al., 2002), leads to substantially higher amounts of SHMT protein products which accumulate in the cytoplasm compared to the reference strain, which contains a single genomic copy of *glyA* (Pasini et al., 2016).

The maximal FucA specific productivity, both in terms of activity and mass, was highest for the reference strain, being 62.04 ± 0.26 AU g^–1^ DCW hr^–1^ and 14.95 ± 0.39 mg g^–1^ DCW hr^–1^, respectively (Table 3). Clearly, the first generation strain showed reduced specific productivity of 44.02 ± 0.68 AU g^–1^ DCW hr^–1^ and 7.61 ± 0.17 mg g^–1^ DCW hr^–1^. A slight improvement was obtained in the third generation strain, obtaining a final value of 48.51 ± 0.31 AU g^–1^ DCW hr^–1^ and 8.95 ± 0.39 mg g^–1^ DCW hr^–1^. Nevertheless, this strain showed volumetric productivity in terms of enzymatic activity comparable to the reference strain, despite still being 22% lower in terms of enzyme mass.

**Table 3.** Specific productivity and volumetric productivity for both fuca activity and mg for the four strains presented along this work

	Specific productivity		Volumetric productivity	
*Escherichia coli* strains	AU FucA g^–1^DCW hr^–1^	mgFucA g^–1^ DCW hr^–1^	AU FucA L^–1^ hr^–1^	mgFucA L^–1^ hr^–1^
M15[pREP4] (reference strain)	62.04 ± 0.26	14.95 ± 0.39	2536 ± 44	611 ± 24
M15Δ*glyA*[pREP4] (first generation strain)	44.02 ± 0.68	7.61 ± 0.17	1652 ± 13	241 ± 80
Puzzle (second generation strain)	45.46 ± 0.26	7.86 ± 0.18	2328 ± 67	407 ± 19
AmpR^–^(third generation strain)	48.51 ± 0.31	8.95 ± 0.39	2597 ± 56	479 ± 26

Unlike the first generation, previous results in shake flasks showed that FucA production improved in the second and third generation expression strains, both of which have a single plasmid, compared to the reference (Pasini et al., 2016). The results obtained in shake flask were consistent with the results presented in this study in bioreactor cultures where the maximum FucA specific production and specific activity increased up to 202 ± 18 mg^–1^ FucA g^–1^ DCW and 1176 ± 19 AU g^–1^ DCW for the second generation, and 1322 ± 19 AU g^–1^ DCW with a specific mass of 245 ± 13 mg FucA g^–1^ DCW for the third generation strain (Fig. 2). Comparing these values with those obtained with the reference strain, both the specific activity and the amount of the recombinant protein are comparable.

Remarkably, the *ΔglyA* strain generations based on a single-plasmid expression system allow for an improvement of FucA production levels compared to the two-plasmid system. In particular, the down-regulation of the *glyA* gene is a key factor that is likely to contribute to improve the regulation of *fucA* expression, thus leading to a higher FucA specific activity. These conclusions are in accordance with the results obtained with the third generation strain. When comparing the first and the third generations, FucA specific activity increased more than 60% (from 806 ± 12 to 1322 ± 19 AU g^–1^ DCW) and FucA specific mass increased c.a. 50% (from 165 ± 13 to 245 ± 13 mgFucA g^–1^ DCW) (Fig. 2).

### SHMT Production Levels

A potential drawback of using SHMT as an auxotrophic marker is accumulation to high concentrations, leading to metabolic burden, and resultant decreases in biomass and RPP (Vidal et al., 2008; Pasini et al., 2016). The second and third generation strains aimed to reduce SHMT levels by regulating *glyA* expression from the weak, constitutive, J23110 promoter (Pasini et al., 2016).

**Fig. 3.**
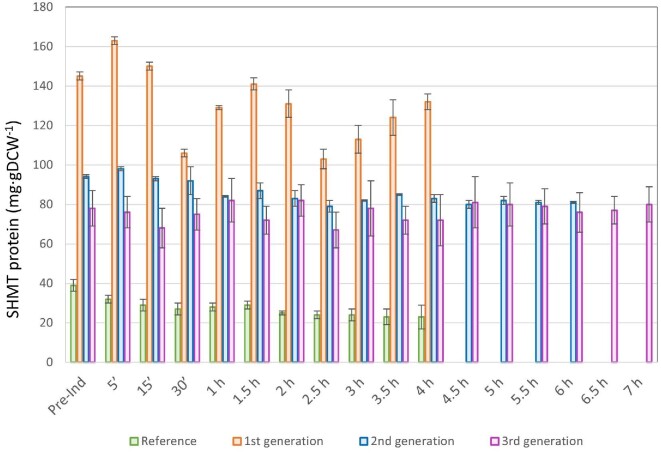
SHMT production (mg g DCW^–1^) for the four strains presented along this study during pre-induction (pre-Ind) and induction phase of high-cell-density fed-batch cultures run in triplicate.

Figure 3 depicts the concentration of the SHMT protein both pre-induction and during the induction phases in the high-cell-density fed-batch cultures carried out in Fig. 1. It can be seen how the SHMT levels are around 130 mg SHMT g^–1^DCW in the first generation strain, which corresponds to a c.a. 4.5-fold increase compared to the reference system (20 mg SHMT g^–1^ DCW). The constitutive high SHMT production can be clearly observed in Supplementary data, Fig. S1A, which as an example presents the SDS-PAGE analysis of the samples taken during the induction phase of the first generation fed-batch culture. Hence, it is clear that the *glyA* gene encoded on a high-copy number plasmid leads to substantially higher amounts of SHMT accumulated in the cytoplasm compared to the reference strain containing a single genomic copy of *glyA*.

However, by tuning *glyA* expression levels, SHMT production decreased in the single-plasmid constructs (second and third generations), resulting in an overall reduced metabolic burden (Pasini et al., 2016). As it can be seen in Fig. 3 and Supplementary data, Fig. S1B, even though there is constitutive overexpression of the SHMT protein in both pre-induction and induction phases, for the second and third generation strains, these values were almost 50% reduced compared to the first generation strains. Thus, these data confirm that the reduction of SHMT production is observed in both bioreactors and shake flasks. As an explanatory example, 85 ± 0.5 and 72 ± 13 mg SHMT g DCW^–1^ were produced after 4 hr of induction for the second and third generation strains, respectively. In contrast, 132 ± 4 mg SHMT g DCW^–1^ was produced for the reference strain (Fig. 3). These findings are equivalent to a 1.6-fold reduction in the accumulation of the SHMT protein in the cytoplasm as a result of replacing the strong P3 promoter with the weaker J23110 constitutive promoter (Pasini et al., 2016). Therefore, this enabled a reduction of the metabolic burden imposed to the cells. These results are in accordance with the observations by Mairhofer et al., (2013), who demonstrated that the folding machinery is severely overstrained in plasmid-based expression systems compared with plasmid-free cells (i.e., with a single copy of the gene of interest integrated into the genome) and this is due to different expression vector dosage.

As stated earlier, similarities can be observed among all the strains when comparing the fed-batch culture results presented here with the previous experiments performed in shake flask cultures (Pasini et al., 2016): (i) the reference strain presents a slightly higher maximum specific growth rate compared to the new set of engineered strains; (ii) the SHMT production increased significantly when moving from the reference to the to the first generation strain; (iii) tuning *glyA* levels is a key factor to allow for improved fucA expression regulation, leading to a higher FucA specific activity in the second and third generations strains; (iv) the second and third generation strains presented a reduction in the amount of acetate production and glucose accumulation.

### Flow Cytometry Study of Physiological Responses Caused by FucA Expression

To gain new insights into the physiology of the strains upon FucA overexpression, FCM analysis was performed on the high-cell-density fed-batch culture experiments performed in Fermac 310/60 bioreactors. The time profiles of the biomass, pH, pO_2_, CO_2_, O_2_, and RQ of the four expression systems evaluated in these bioreactors are represented as an example in Supplementary data, Fig. S2. Importantly, pO_2_ time profile between the set of experiments carried out in Biostat® B and the Fermac 310/60 bioreactors were similar.

**Fig. 4.**
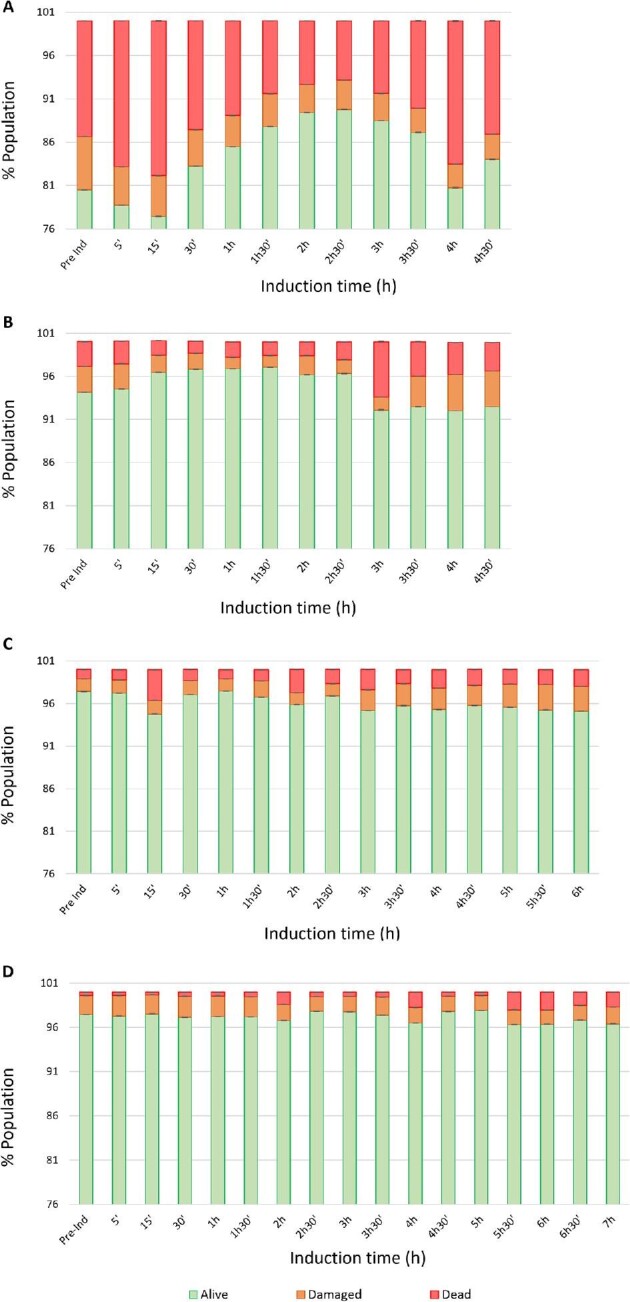
BOX/PI co-staining, indicating the percentage of (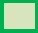) live (PI^–^/BOX^–^), (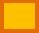) damaged (PI^–^/BOX^+^), and (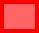) dead (PI^+^/BOX^+^) cells for the four expression systems presented along this study during pre-induction (pre-Ind) and induction phase (from 5 to 7 hr) of duplicate high-cell-density fed-batch cultures using the Fermac 310/60 bioreactor. (A) The reference strain; (B) the first generation; (C) the second generation; and (D) the third generation.

Propidium iodide (PI), which only stains cells with compromised membranes, and bis-oxanol (BOX), which only stains cells with a collapsed membrane potential, were used to determine viability (Fig. 4). Cells were classified as ‘‘healthy’’ with a membrane potential (PI^–^/BOX^–^), ‘‘damaged’’, being intact but with no membrane potential (PI^–^/BOX^+^), or ‘‘dead’’, having a non-intact membrane and no membrane potential (PI^+^/BOX^+^).

As can be seen in Fig. 4A, from the pre-induction sample to the end of the fermentation, the reference strain presented a significant percentage of unhealthy and dead cells even under uninduced conditions. The high proportion of damaged or dead cells present before the induction stage is probably due to the presence of the antibiotic in the medium, which is used to maintain plasmid stability (Feizollahzadeh et al., 2017). The percentage of damaged cells remained relatively constant through growth (3–5%). The percentage of dead cells reached the maximum value (18%) after 15 min of induction, which indicates the stress caused to the cells by the induction of FucA expression.

**Fig. 5.**
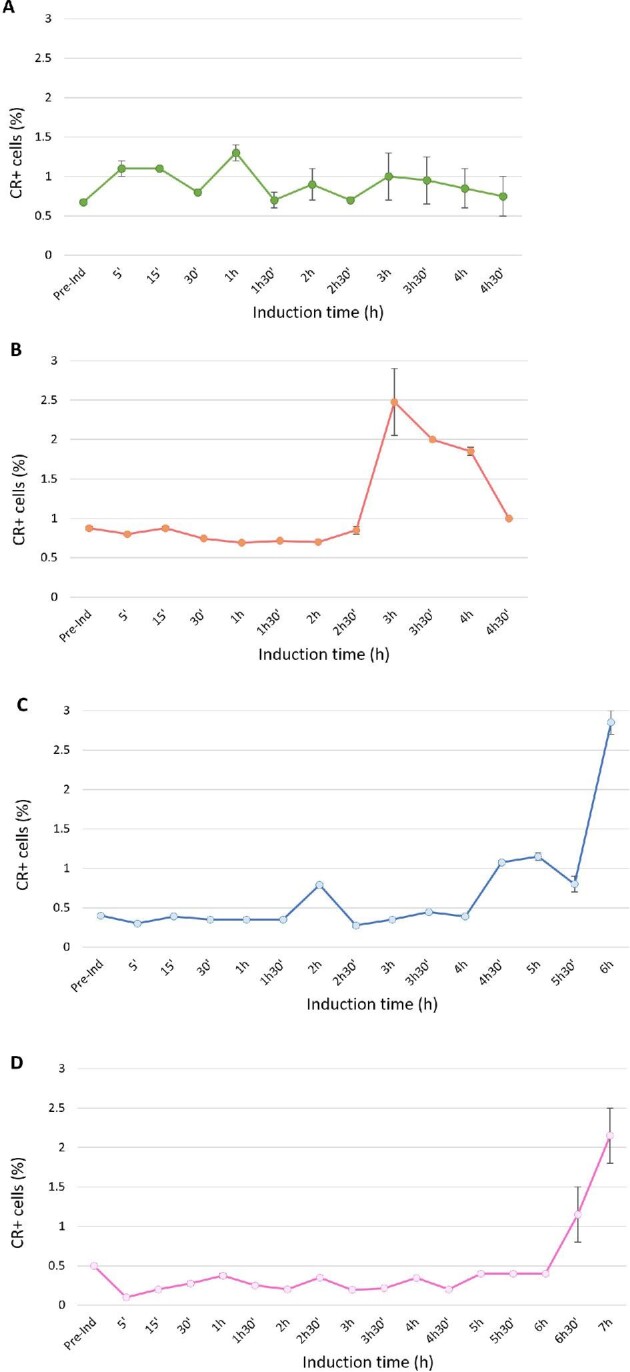
Time profiles of the percentage of Congo red-stained cells as determined by FCM. (A) The reference strain; (B) the first generation; (C) the second generation; and (D) the third generation. Average values and error bars are presented from two independent experiments.

For the two-plasmid based expression systems (the reference and the first generation strains), it can be seen that at the end of the fed-batch cultures, the proportion of damaged and dead cells increased (Fig. 4A and B). It is well known that the production of a foreign protein causes additional stress for the host strain and leads to a decrease in the overall cell fitness (Hoffmann & Rinas, 2004). The dead population at the end of the fermentation corresponded to 16% and 8% for the reference and the first generation strains, respectively. This reflects the stress caused to the cells due to the induction. The state of the cells of the reference strain is more unhealthy than the first generation strain, probably due to a higher FucA overexpression and also due to the presence of the antibiotic. In addition to PI&BOX staining, we also stained cells with CR, which stains amyloids such as IBs (Carrió et al., 2005). Figure 5A and B shows that, for the reference and first generation strains, there were around 1% of cells containing IBs; for the first generation strain, this increased after 3 hr of induction.

FCM data reveal that for the second generation strain, Puzzle, more than 95% of cells were healthy throughout the fermentation (Fig. 4C). Similar observations were obtained for the third generation strain (AmpR^–^) (Fig. 4D), with even fewer unhealthy cells (96% healthy throughout). Almost no change in PI or BOX staining was observed through the induction phase for the second and third generation strains, indicating that physiology was not negatively impacted by RPP.

Only at the very end of the fermentation a slight increase of both damaged and dead cells appears, for the Puzzle and the AmpR^–^ strains, indicating an initial loss of viability. This is in accordance with previous works, where the number of cells identified as either being stressed or dead increased at the end of the fermentation, due to stress condition (Lewis et al., 2004). This increase is probably due to the non-optimal conditions presented at the end of the fermentation, in particular the low pO_2_ levels due to the high oxygen demands of the culture (data not shown). This stress condition can also be observed in the FCM analysis of cells stained with CR (Fig. 5C and D), showing an increase in the proportion of cells containing IBs (up to 2% and 3% of cells, respectively). This result can be explained by an increase of the stress condition of the protein folding machinery resulting in an increase of aggregated protein. This can be reflected also in the specific enzyme activity values of the strains presented in this study, being: 5.06 ± 0.01 UA mg^–1^ for the reference strain; 4.88 ± 0.01 UA mg^–1^ for the first generation; 5.82 ± 0.01 UA mg^–1^ for the second generation; and 5.40 ± 0.01 UA mg^–1^ for the third generation. These results indicate that the first generation strain presents the lower FucA enzyme activity/protein concentration ratio indicating a lower folded enzyme activity, in accordance with the CR results that indicate the higher amount of IBs. It is interesting to note that the second generation strain showed the higher specific enzyme activity.

Bacterial exposure to environmental stress (including glucose/oxygen availability) often triggers protein misfolding and the formation of aggregates as IBs (Kwiatkowska et al., 2008; Schramm et al., 2020). These results are consistent with those presented by Castellanos-Mendoza et al., where IB formation (as detected using CR) increased at uncontrolled pH conditions (Castellanos-Mendoza et al., 2014).

The presence of IBs is generally not desirable in the recombinant production of enzymes because a further refolding step during purification is often required (Vallejo & Rinas, 2004). In future studies, a trade-off between maximizing protein production and correct folding of the enzyme should be taken into consideration if further downstream processing—harvesting, purification, and application of FucA—is intended (Mukhopadhyay, 1997; Kim at el., 2020).

## Conclusion

We have demonstrated that by tuning the expression levels of the repressor (*lacI)* and the auxotrophic gene (*glyA)* using synthetic biology tools and methods, the strains developed in our lab can be used for efficient and stable expression of an industrially relevant enzyme, namely, FucA, in cultivation conditions that resemble industrial production settings. FucA production (mg FucA g DCW^–1^) in the optimized third generation strain was c.a. 5%, 50%, and 10% higher comparing with the reference, first generation, and second generation strains, respectively. FucA activity (AU g^–1^DCW) was increased through the different stepwise improvements performed along this work. The best performing engineered strain reached more than 60% higher values in terms of activity compared to the first generation of M15Δ*glyA* strain.

Tuning of *glyA* transcriptional levels using the weak constitutive promoter J23110 resulted in the reduction of more than 50% of SHMT expression levels in the single plasmid expression system strains, which led to a reduction of the energy demand toward SHMT production, thus redirecting nutrients/energy for recombinant FucA production.

To conclude, the optimized third generation strain presented a reduction of the metabolic burden and an improvement of the strain fitness and overall performance of the expression. The expression levels were similar to the commercial strain, but our novel expression system presented advantageous features: (i) it is a single-plasmid tightly regulated expression system; (ii) it presented significant reduced metabolic burden to the cells, resulted in no acetate production nor glucose accumulation and in healthy cells with intact and polarized cytoplasmatic membrane all along the fermentation; and (iii) no antibiotic is needed.

Remarkably, all this is achieved by means of (i) a system with a reduced number of genetic components and, importantly, (ii) antibiotic-free, which makes it suitable for biopharmaceutical and agritech applications.

We envisage that our antibiotic-free expression system has the potential to become an attractive platform for the production of a wide range of proteins as well as for other applications, where the use of antibiotics is restricted, such as vaccine delivery (Saubi et al., 2014).

## Supplementary Material

kuac018_Supplemental_File

## References

[bib1] Al-Allaf F. A. , Tolmachov O. E., Zambetti L. P., Tchetchelnitski V., Mehmet H. (2013). Remarkable stabilityu of an instability-prone lentiviral vector plasmid in *Escherichia coli* Stbl3. 3 Biotech, 3(1), 61–70. 10.1007/s13205-012-0070-8. PMC3563744 28324350

[bib2] Ammar E. M. , Wang X., Rao C. V. (2018). Regulation of metabolism in *Escherichia coli* during growth on mixtures of the non-glucose sugars: arabinose, lactose, and xylose. Scientific Reports, 8(1), 609. 10.1038/s41598-017-18704-0. 29330542 PMC5766520

[bib4] Brown T. A. (1995) Gene cloning: an introduction (4th edition), Wiley-Blackwell.

[bib5] Calleja D. , Fernández-Castañé A., Pasini M., de Mas C., López-Santín J. (2014). Quantitative modeling of inducer transport in fed-batch cultures of *Escherichia coli*. Biochemical Engineering Journal, 91, 210–219. 10.1016/j.bej.2014.08.017.

[bib6] Carneiro S. , Ferreira E. C., Rocha I. (2013). Metabolic responses to recombinant bioprocesses in *Escherichia coli*. Journal of Biotechnology, 164(3), 396–408. 10.1016/j.jbiotec.2012.08.026. 23022453

[bib7] Carrió M. , Gonzalez-Montalbán N., Vera A., Villaverde A., Ventura S. (2005). Amyloid-like properties of bacterial inclusion bodies. Journal of Molecular Biology, 347(5), 1025–1037. 10.1016/j.jmb.2005.02.030. 15784261

[bib8] Castellanos-Mendoza A. , Castro-Acosta R. M., Olvera A., Zavala G., Menodza-Vera M., Garcia-Hernandez E., Alagon A., Trujillo-Roldan M. A., Valdez-Cruz N. A. (2014). Influence of pH control in the formaiton of inclusion bodies during production of recombinant sphyingomyelinase-D in *Escherichia coli*. Microbial Cell Factories, 13(1), 137. 10.1186/s12934-014-0137-9. 25213001 PMC4177172

[bib9] Castiñeiras T. S. , Williams S. G., Hitchcock A. G., Smith D. C. (2018). *E. coli* strain engineering for the production of advanced biopharmaceutical products. FEMS Microbiology Letters, 365(15), 162. 10.1093/femsle/fny162. 29982628

[bib10] Clapés P. , Fessner W. D., Sprenger G. A., Samland A. K. (2010). Recent progress in stereoselective synthesis with alsolases. Current Opinion in Chemical Biology, 14(2), 154–167. 10.1016/j.cbpa.2009.11.029. 20071212

[bib11] Costa S. , Almeida A., Castro A., Domingues L. (2014). Fusion tags for protein solubility, purification, and immunogenicity in Escherichia coli: the novel Fh8 system. Frontiers in Microbiology, 5(5), 63. 10.3389/fmicb.2014.00063. 24600443 PMC3928792

[bib12] Durany O. , de Mas C., López-Santín J. (2005). Fed-batch production of recombinant fuculose-1-phosphate aldolase in *E. coli*. Process Biochemistry, 40(2). 707–716. 10.1016/j.procbio.2004.01.058.

[bib13] Feizollahzadeh S. , Kouhpayeh S., Rahimmansh I., Khanahmad H., Sabzehei F., Ganjalikhani-hakemi M., Andalib A., Hejazi Z., Razaei A. (2017). The increase in preotein and palmsid yields of *E. coli* with optmized concentration of Ampicillin as selection marker. Iranian Journal of Biotechnology, 15(2), 128–134. 10.15171/ijb.1467. 29845060 PMC5811054

[bib14] Fernández-Castané A. , Caminal G., López-Santín J. (2012). Direct measurement of IPTG enable analysis of the induction of *E. coli* in high cell density cultures. Microbial Cell Factories, 11( 1), 58. 10.1186/1475-2859-11-58. 22571410 PMC3442970

[bib15] Fernández-Castané A. , Li H., Thomas O. R. T., Overton T. W. (2017). Flow cytometry as a rapid analytical tool to determine physiological responses to changin O_2_ and iron concentration by *Magnetospirillum gryphiswaldense* strain MSR-1. Scientific Reports, 7(1), 13118. 10.1038/s41598-017-13414-z. 29030621 PMC5640647

[bib16] Fernández-Castané A. , Vine C. E., Caminal G., López-Santín J. (2012). Evidencing the role of lactose permease in IPTG uptake by *Escherichia coli* in fed-batch high cell density cultures. Journal of Biotechnology, 157(3). 391–398. 10.1016/j.jbiotec.2011.12.007. 22202176

[bib17] Garcia-Junceda E. , Gwo-Jenn S., Takeshi S., Wong C. H. (1995). A new strategy for the cloning, overexpression and one step purification of Tthree DHAP-dependent aldolases: rhamnulose-1-phosphate aldolase, fuculose-1-phosphate aldolase and tagatose-1,6-diphosphate aldolase. Bioorganic & Medicinal Chemistry, 3(7), 945–953. 10.1016/0968-0896(95)00077-t. 7582972

[bib18] Germán L. R. , Morales E. S., Ceccarelli E. A. (2019). New tools for recombinant protein production in *Escherichia coli*: a 5-year update. Protein Science, 28(8). 1412–1422. 10.1002/pro.3668. 31219641 PMC6635841

[bib19] Glick B. R. (1995). Metabolic load and heterologous gene expression. Biotechnology Advances, 13(2), 247–261. 10.1016/0734-9750(95)00004-A. 14537822

[bib20] Glenting J. , Wessels S. (2005). Ensuring safety of DNA vaccines. Microbial Cell Factories, 4(1), 26. 10.1186/1475-2859-4-26. 16144545 PMC1215512

[bib21] Gronenborn B. (1976). Overproduction of phage lambda repressor under control of the lac promotor of *Escherichia coli*. Molecular and General Genetics MGG, 148(3), 243–250. 10.1007/BF00332898. 796661

[bib22] Hewitt C. J. , Nebe-von Caron G., Nienow A. W., Mcfarlane C. M. (1999). The use of multi-parameter flow cytometry to compare the physiological response of *Escherichia coli* w3110 to glucose limitation during batch, fed-batch and continuous culture cultivations. Journal of Biotechnology, 75(2-3), 251–264. 10.1016/s0168-1656(99)00168-6. 10553662

[bib23] Hoffmann F. , Rinas U. (2004). Stress induced by recombinant protein production in *Escherichia coli*. Advances Biochemical Engineering Biotecnology, 89, 73–92. 10.1007/b93994. 15217156

[bib24] Jeong K. J. , Lee S. Y. (2003). Enhanced production of recombinant proteins in *Escherichia coli* by filamentation suppression. Applied and Environmental Microbiology, 69(2), 1295–1298. 10.1128/AEM.69.2.1295-1298.2003. 12571061 PMC143674

[bib25] Kim J. , Darlington A., Salvador M., Utrilla J., Jimenez J. I. (2020). Trade-offs between gene exession, growth and phenotypic diversity in microbial populations. Current Opinion in Biotechnology, 62, 29–37. 10.1016/j.copbio.2019.08.004. 31580950 PMC7208540

[bib26] Kazan D. , Çamurdan A., Hortaçsu A. (1995). The effect of glucose concentration on the growth rate and some intracellular components of a recombinant *E. coli* culture. Process Biochemistry, 30(3), 269–273. 10.1016/0032-9592(95)85008-2.

[bib27] Koeller K. M. , Wong C. H. (2000). Caomplex carbohydrate synthesis tools for glycobiologists: enzyme-based approach and programmable one-pot strategies. Glycobiology, 10(11), 1157–1169. 10.1093/glycob/10.11.1157. 11087708

[bib28] Kwiatkowska J. , Matuszewska E., Kuczyńska-Wiśnik D., Laskowska E. (2008). Aggregation of *Escherichia coli* proteins during stationary phase depends on glucose and oxygen availability. Research in Microbiology, 159(9-10), 651–657. 10.1016/j.resmic.2008.09.008. 18983914

[bib29] Lartigue M. F. , Leflon-Guibout W., Poirel L L., Nordmann P., Nicolas-Chanoine M. H. (2002). HPromoters P3, Pa/Pb, P4, and P5 upstream from bla(TEM) genes and their relationship to beta-lactam resistance. Antimicrobial Agents and Chemotherapy, 46(12), 4035–7. 10.1128/AAC.46.12.4035-4037. 12435720 PMC132779

[bib30] Lewis G. , Taylor I. W, Nienow A. W., Hewitt C. J. (2005). The application of multi-parameter flow cytometry to the study of recombinant *Escherichia coli* batch fermentation processes. Microbial Cell Factories, 4(1), 311–322. 10.1186/1475-2859-4-1. 15249970

[bib31] Liu M. , Feng X., Ding Y., Zhao G., Liu H., Xian M. (2015). Metabolic engineering of *Escherichia coli* to improve recombinant protein production. Applied Microbiology and Biotechnology, 99(24), 10367–10377. 10.1007/s00253-015-6955-9. 26399416

[bib32] Mairhofer J. , Scharl T., Marisch K., Cserjan-Puschmann M., Striedner G. (2013). Comparative transcription profiling and in-depth characterization of plasmid-based and plasmid-free *Escherichia coli* expression systems under production conditions. Applied and Environmental Microbiology, 79(12), 3802–3812. 10.1128/AEM.00365-13. 23584782 PMC3675926

[bib33] Marschall L. , Segmeister P., Herwig C. (2017). Tunable recombiant protein expression in *E. coli* promoter systems and genetic constraints. Applied Microbiology and Biotechnology, 101(2), 501–512. 10.1007/s00253-016-8045-z. 27999902 PMC5566544

[bib34] Mignon C. , Sodoyer R., Werle B. (2015). Antibiotic-free selection in biotherapeutics: now and forever. Pathogens, 4(2), 157–181. 10.3390/pathogens4020157. 25854922 PMC4493468

[bib35] Mukhopadhyay A. (1997). Inclusion bodies and purification of proteins in biologically active forms. Advances in Biochemical Engineering/Biotechnology, 56. 61–109. 10.1007/BFb0103030. 8939059

[bib36] Neubauer P. , Lin H. Y., Mathiszik B. (2003). Metabolic load of recombinant protein production: inhibition of cellular capacities for glucose uptake and respiration after induction of a heterologous gene in *Escherichia coli*. Biotechnology and Bioengineering, 83(1), 53–64. 10.1002/bit.10645. 12740933

[bib37] Pasini M. , Fernández-Castané A., Jaramillo A., De Mas C., Caminal G., Ferrer P. (2016). Using promoter libraries to reduce metabolic burden due to plasmid-encoded proteins in recombinant *Escherichia coli*. New Biotechnology, 33(1), 78–90. 10.1016/j.nbt.2015.08.003. 26335036

[bib38] Phue J.-N. , Shiloach J. (2005). Impact of dissolved oxygen concentration on aceate accumulation and physiology of *E. coli* BL21, evaluating transciption levels of key genes at different dissolved oxygen conditions. Metabolic Engineering, 7(5-6), 353–363. 10.1016/j.ymben.2005.06.003. 16099189

[bib39] Pierce J. , Gutteridge S. (1985). Large-scale preparation of ribulosebisphosphate carboxylase from a recombinant system in *Escherichia coli* characterized by extreme plasmid instability. Appl Env Microbiol, 49(5). 1094–1100. 10.1128/aem.49.5.1094-1100. 3890745 PMC238512

[bib40] Pinsach J. , de Mas C., López-Santín J. (2008). Induction strategies in fed-batch cultures for recombinant protein production in *Escherichia coli*: application to rhamnulose 1-phosphate aldolase. Biochemical Engineering Journal, 41(2), 181–187. 10.1016/j.bej.2008.04.013.

[bib41] Plamann M. D. , Stauffer G. V. (1983). Characterisation of the *Escherichia coli* gene for serine hydroxymethyltransferase. Gene, 22(1), 9–18. 10.1016/0378-1119(83)90059-8. 6190704

[bib42] Rahmen N. , Fulton A., Ihling N., Magni M., Jaeger K., Büchs J. (2015). Exchange of single amino acids at different positions of a recombinant protein affects metabolic burden in *Escherichia coli*. Microbial Cell Factories, 14(1). 10.1186/s12934-015-0191-y. PMC4307990 25612616

[bib43] Rosano G. L. , Ceccarelli E. A. (2014). Recombinant protein expression in *Escherichia coli*: advances and challenges. Frontiers in Microbiology, 5. 172. 10.3389/fmicb.2014.00172. 24860555 PMC4029002

[bib44] Saubi N. , Gea-Mallorquí E., Ferrer P., Hurtado C., Sánchez-Úbeda S., Eto Y., Gatell J. M., Hanke T., Joseph J. (2014). Engineering new mycobacterial vaccine design for HIV-TB pediatric vaccine vectored by lysine auxotroph of BCG. Molecular Therapy - Methods & Clinical Development, 1. 14017. 10.1038/mtm.2014.17. 26015961 PMC4362382

[bib45] Schramm F. D , Schroeder K., Jonas K. (2020). Protein aggregation in bacteria. FEMS Microbiology Reviews, 44(1). 54–72. 10.1093/femsre/fuz026. 31633151 PMC7053576

[bib46] Sooan S. , Chang D., Pan J. G. (2009). Acetate consuption activity directly determines the level of acetate accumulation during *Escherichia coli* W3110 growth. Journal of Microbiology and Biotechnology, 19(10), 1127–1134. 10.4014/jmb.0902.0097. 19884769

[bib47] Vallejo L. F. , Rinas U. (2004). Strategies for the recovery of active proteins through refolding of bacterial inclusio body proteins. Microbial Cell Factories, 3(1), 11. 10.1186/1475-2859-3-11. 15345063 PMC517725

[bib48] Vidal L. , Durany O., Suau T., Ferrer P., Caminal G. (2003). High-level production of recombinant His-tagged rhamnulose 1-phosphate aldolase in *Escherichia coli*. Journal of Chemical Technology & Biotechnology, 78(11), 1171–1179.

[bib49] Vidal L. , Ferrer P., Alvaro G., Benaiges M. D., Caminal G. (2005). Influence of induction and operation mode on recombinant rhamnulose 1-phosphate aldolase production by *Escherichia coli* using the T5 promoter. Journal of Biotechnology, 118(1), 75–87. 15908029 10.1016/j.jbiotec.2005.02.012

[bib50] Vidal L. , Pinsach J., Striedner G., Ferrer P. (2008). Development of an antibiotic-free plasmid selection system based on glycine auxotrophy for recombinant protein overproduction in *Escherichia coli*. Journal of Biotechnology, 134(1-2), 127–136. 10.1016/j.jbiotec.2008.01.011. 18308411

[bib51] Villarejo M. R. , Zabin I. (1974). β -galactosidase from termination and deletion mutant strains. Journal of Bacteriology, 120(1). 466–474. 10.1128/jb.120.1.466-474.1974. 4607501 PMC245784

[bib52] Waegeman H. , Soetaert W. (2011). Increasing recombinant protein production in *Escherichia coli* through metabolic and genetic engineering. Journal of Industrial Microbiology & Biotechnology, 38(12), 1891–1910. 10.1007/s10295-011-1034-4. 21901404

[bib53] Weber J. , Hoffmann F., Rinas U. (2002). Metabolic adaptation of *Escherichia coli* during temperature-induced recombinant protein production: 2. Redirection of metabolic fluxes. Biotechnology and Bioengineering, 80(3), 320–330. 10.1002/bit.10380. 12226865

[bib54] Weber J. , Li. Z., Rinas U. (2021). Recombinant protein production provoked accumulation of ATP, fructose-1,6-bisphosphate and pyruvate in *E. coli* K12 strain TG1. Microbial Cell Factories, 20(1), 169. 10.1186/s12934-021-01661-9. 34446023 PMC8394631

[bib55] Wyre C. , Overton T. W. (2014a). Flow cytometric analysis of *E. coli* on agar plates: implications for recombinant protein production. Biotechnology Letters, 36(7), 1485–1494. 10.1007/s10529-014-1511-8. 24652548

[bib56] Wyre C. , Overton T. W. (2014b). Use of a stress-minimisation paradigm in high cell density fed-batch *Escherichia coli* fermentations to optimise recombinant protein production. Journal of Industrial Microbiology and Biotechnology, 41(9), 1391–1404. 10.1007/s10295-014-1489-1. 25056840

[bib57] Xu J. , Matthews K. S. (2009). Flexibility in the inducer binding region is crucial for allostery in the *Escherichia coli* lactose repressor. Biochemistry, 48(22), 4988–4998. 10.1021/bi9002343. 19368358 PMC2772868

[bib58] Zamenhof P. J. , Villarejo M. (1972). Construction and properties of *Escherichia coli* strains exhibiting α-complementation of β-galactosidase fragments in vivo. Journal of Bacteriology, 110(1), 171. 10.1128/jb.110.1.171-178.1972. 4552986 PMC247395

