# Supplementary material for: Process intensification at the expression system level for the production of 1-phosphate aldolase in antibiotic-free E. coli fed-batch cultures
Source: J Ind Microbiol Biotechnol. 2022 Jun 6;49(4):kuac018. doi: 10.1093/jimb/kuac018 (PMC9339150; doi:10.1093/jimb/kuac018)
Supplement: kuac018_Supplemental_File [file kuac018_supplemental_file.docx]

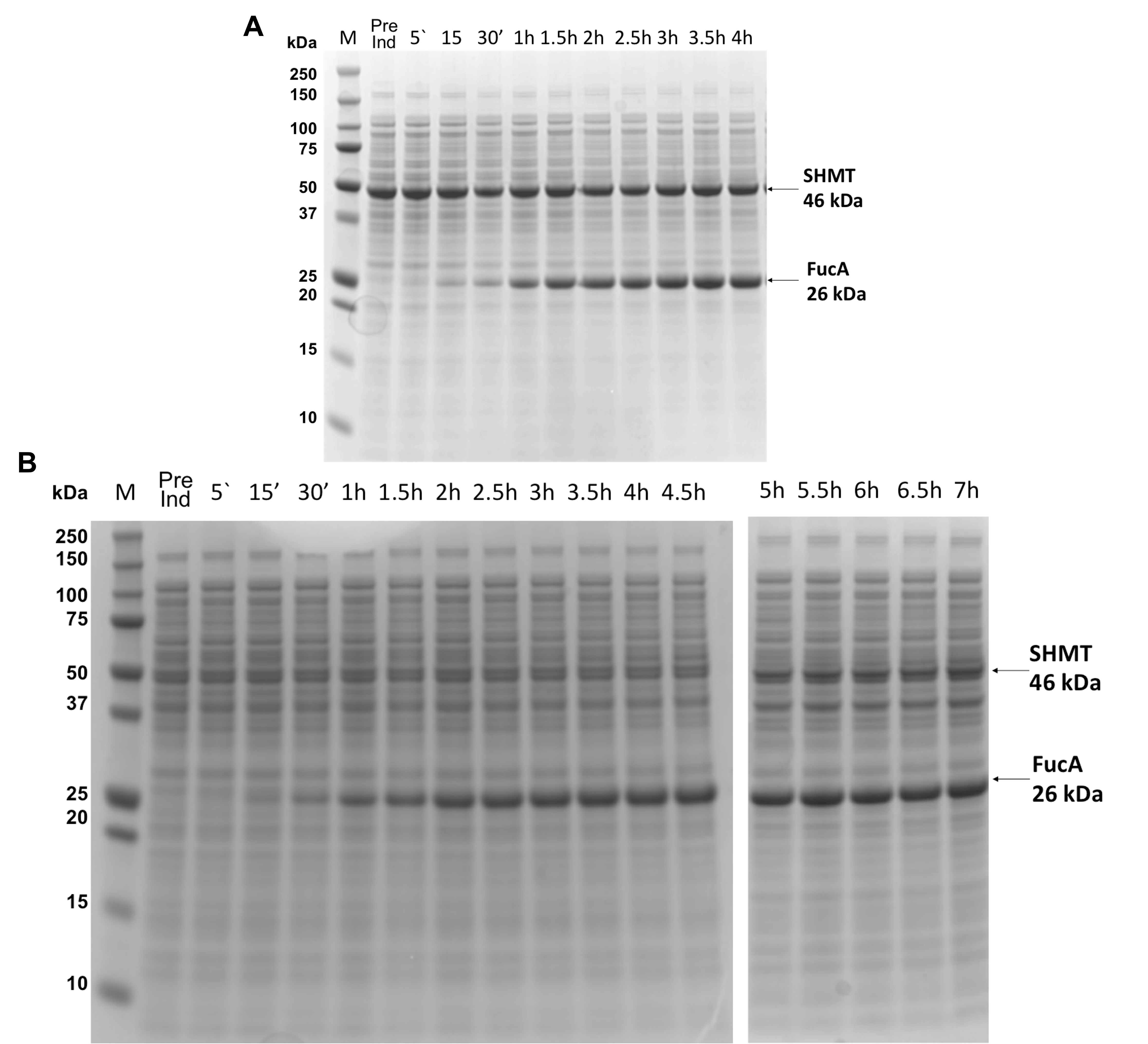


Figure S1 SDS-PAGE of Fed-Batch culture: [IPTG], 70 μM; X_ind_, 20 g·L^-1^; μ 0.22 h^-1^ of A) 1^st^ generation two-plasmids expression system, M15Δ*glyA*[pREP4] and B) 3^rd^ generation single plasmid expression system, AmpR^-^. Pre Ind, pre-induction; from 5’ to 7 h correspond to the time after induction. The 26 kDa FucA and the 46 kDa SHMT are indicated in the figure.


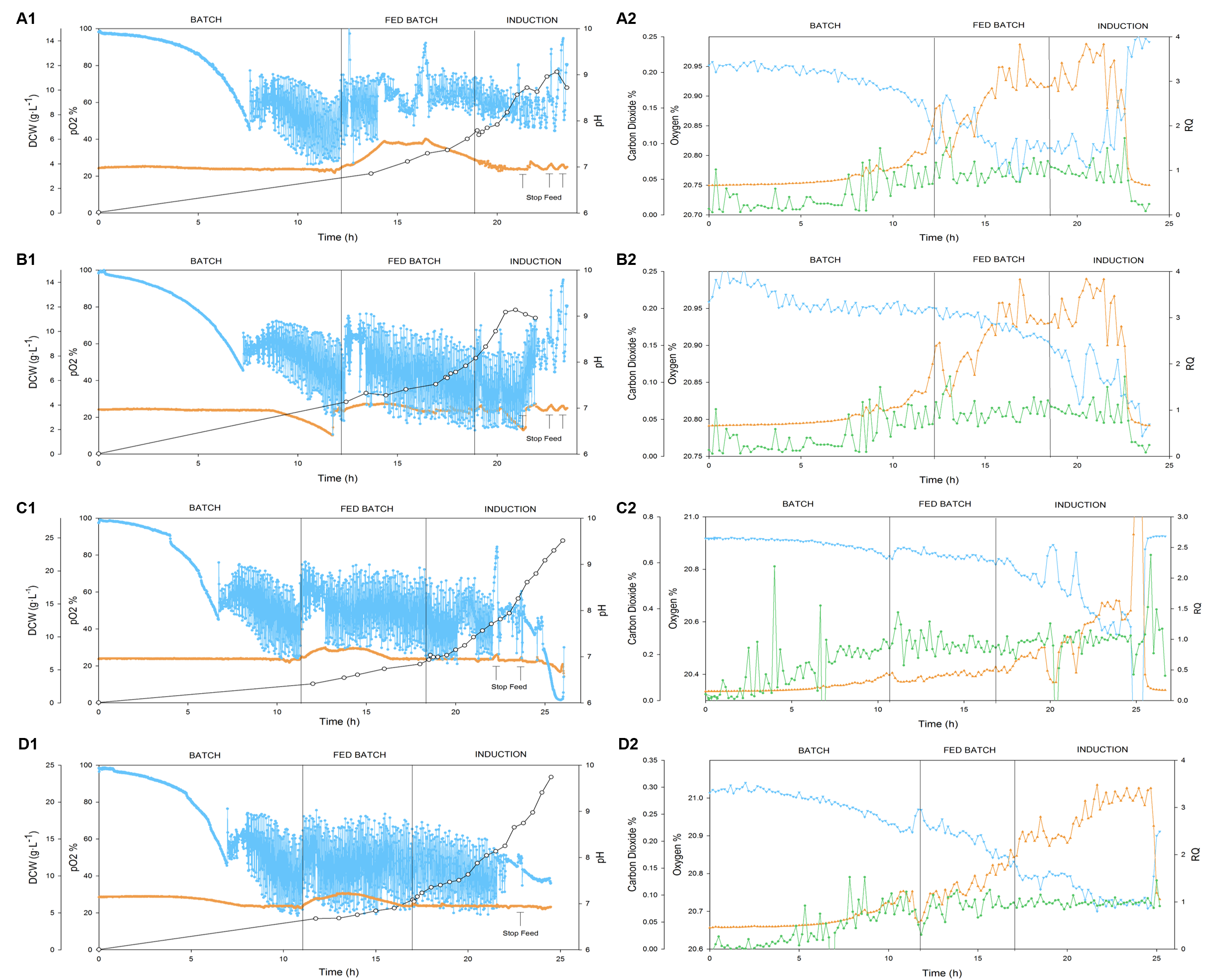


Figure S2 *E. coli* fed-batch culture for the four expression systems presented along this study: [IPTG], 70 μM; X_ind_, 6 g·L^-1^; μ 0.22 h^-1^. 1) pO_2_ in light blue, pH in orange, (🞆) Biomass DCW (g·L^-1^) along time. Batch, fed-batch and induction phases are indicated. The arrows indicate the stop of the feeding. 2) Gas-MS data for offgas: % of oxygen (O_2_) in light blue, % of carbon dioxide in orange (CO_2_) and the respiratory quotient (RQ) in green. (A) Reference strain; (B) 1^st^ generation; (C) 2^nd^ generation; (D) 3^rd^ generation. The partial pressure of oxygen (pO_2_) decreases along the batch phase exponentially up to the set point of 50 %, where an agitation controller is started in order to maintain the level.
